# Recent Progress in Chitosan-Based Nanomedicine for Its Ocular Application in Glaucoma

**DOI:** 10.3390/pharmaceutics15020681

**Published:** 2023-02-17

**Authors:** Hassan A. Albarqi, Anuj Garg, Mohammad Zaki Ahmad, Abdulsalam A. Alqahtani, Ismail A. Walbi, Javed Ahmad

**Affiliations:** 1 Department of Pharmaceutics, College of Pharmacy, Najran University, Najran 11001, Saudi Arabia; 2 Institute of Pharmaceutical Research, GLA University, Mathura 281406, India; 3 Department of Clinical Pharmacy, College of Pharmacy, Najran University, Najran 11001, Saudi Arabia

**Keywords:** glaucoma, chitosan, nanoparticles, mucoadhesion, ocular bioavailability, therapeutic efficacy

## Abstract

Glaucoma is a degenerative, chronic ocular disease that causes irreversible vision loss. The major symptom of glaucoma is high intraocular pressure, which happens when the flow of aqueous humor between the front and back of the eye is blocked. Glaucoma therapy is challenging because of the low bioavailability of drugs from conventional ocular drug delivery systems such as eye drops, ointments, and gels. The low bioavailability of antiglaucoma agents could be due to the precorneal and corneal barriers as well as the low biopharmaceutical attributes of the drugs. These limitations can be overcome by employing nanoparticulate drug delivery systems. Over the last decade, there has been a lot of interest in chitosan-based nanoparticulate systems to overcome the limitations (such as poor residence time, low corneal permeability, etc.) associated with conventional ocular pharmaceutical products. Therefore, the main aim of the present manuscript is to review the recent research work involving the chitosan-based nanoparticulate system to treat glaucoma. It discusses the significance of the chitosan-based nanoparticulate system, which provides mucoadhesion to improve the residence time of drugs and their ocular bioavailability. Furthermore, different types of chitosan-based nanoparticulate systems are also discussed, namely nanoparticles of chitosan core only, nanoparticles coated with chitosan, and hybrid nanoparticles of chitosan. The manuscript also provides a critical analysis of contemporary research related to the impact of this chitosan-based nanomedicine on the corneal permeability, ocular bioavailability, and therapeutic performance of loaded antiglaucoma agents.

## 1. Introduction

**Figure 1:**
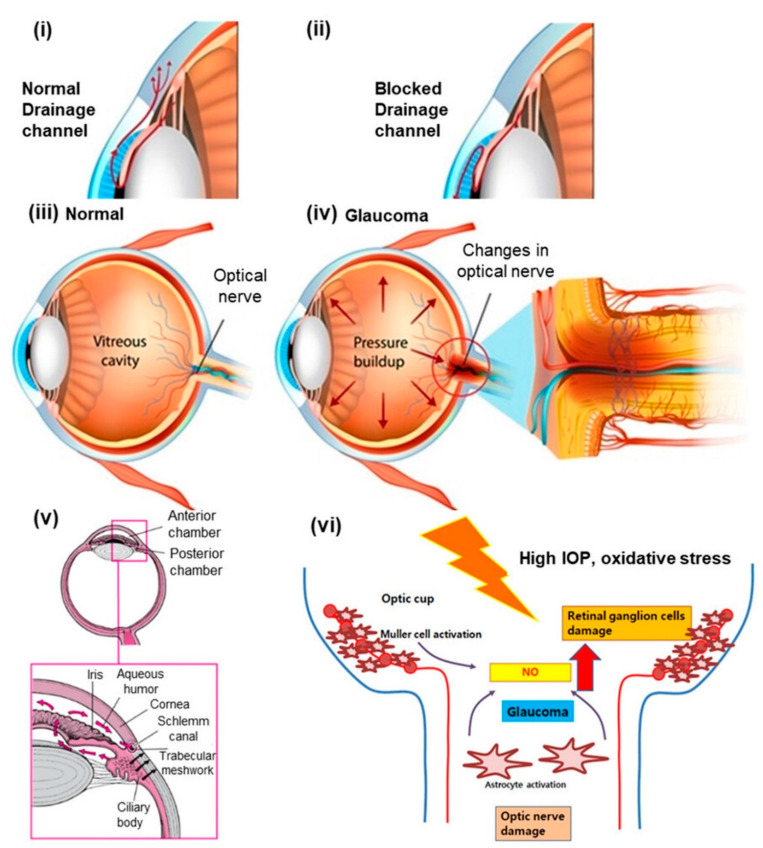
Schematic illustration presenting the pathophysiology of the eye in glaucoma compared to normal eyes. (**i**) Normal drainage channel in healthy eye. (**ii**) Blocked drainage channel in glaucoma. (**iii**) Normal IOP in a vitreous cavity and normal optical nerve in a healthy eye. (**iv**) Rise in IOP in a vitreous cavity and changes in the optical nerve in glaucoma. (**v**) Ocular fluid is produced in a posterior chamber at the ciliary body behind the iris and flows through the anterior chamber of the eye to ultimately come out through the uveoscleral pathway (highlighted by the black arrow). (**vi**) Rise in IOP and oxidative stress in the glaucoma-conditioned eye finally led to damage to retinal ganglion cells and optical nerves. Reproduced from Patel et al. [10], Elsevier, 2022.

Glaucoma is a degenerative disease that requires lifetime drug treatment and can be acute or chronic [1]. A blockage in the flow of aqueous humor between the front and back of the eye causes intraocular pressure to rise quickly, retinal degeneration, and optic neuropathy, all of which are signs of acute glaucoma [2,3]. If left untreated, acute glaucoma can cause permanent vision loss within hours or days. Acute glaucoma is more severe and causes serious visual loss in three times as many people as chronic glaucoma does, even though the former is more common (23.4 vs. 52.7 million occurrences in 2020) [4]. The schematic illustration in Figure 1 represents the pathophysiology of glaucoma. It illustrates the normal mechanism of ocular fluid production and drainage channels for its normal flow compared to the obstruction of flow drainage leading to an increase in intraocular pressure (IOP), ultimately affecting the vision due to optic nerve damage. The ocular fluid is produced in the posterior chamber of the eyes at the ciliary body behind the iris and flows through the anterior chamber of the eye to ultimately come out through the uveoscleral pathway. The rise in IOP and oxidative stress in the glaucoma-conditioned eye finally led to damage to retinal ganglion cells and optical nerves [4,5]. Neurovascular dysfunction and neuroinflammation of the eye have also been implicated in the pathogenesis of glaucoma. It is very well reported that oxidative stress and choroidal vascular dysfunction are mainly involved in the pathogenesis of age-related macular degeneration [2,3]. In addition, the poor bioavailability of presently marketed medications necessitates frequent doses and low patient compliance, putting the vision of patients at further risk. Ninety percent of commercial ocular drugs are available as eye drops, which is a simple and effective way to administer a drug. However, corneal permeability limits the absorption, bioavailability, and therapeutic activity of ocular drugs [6]. Ocular barriers, such as the precorneal, corneal, and conjunctival layers, limit drug diffusion in ocular tissues. The precorneal barriers include blinking reflexes, lacrimal turnover, nasolacrimal drainage, efflux transporters, and drug metabolism by lysozymes present in tears. Furthermore, ocular absorption of drugs is poor due to drug binding to or repulsion from the conjunctiva and tight junctional complexes in the corneal epithelium [7]. In recent years, several novel ocular drug delivery systems were investigated utilizing nanotechnology-mediated drug delivery strategies to overcome the pre-corneal and corneal barriers to enhance the ocular absorption and hence therapeutic efficacy of drugs [8,9].

**Figure 2:**
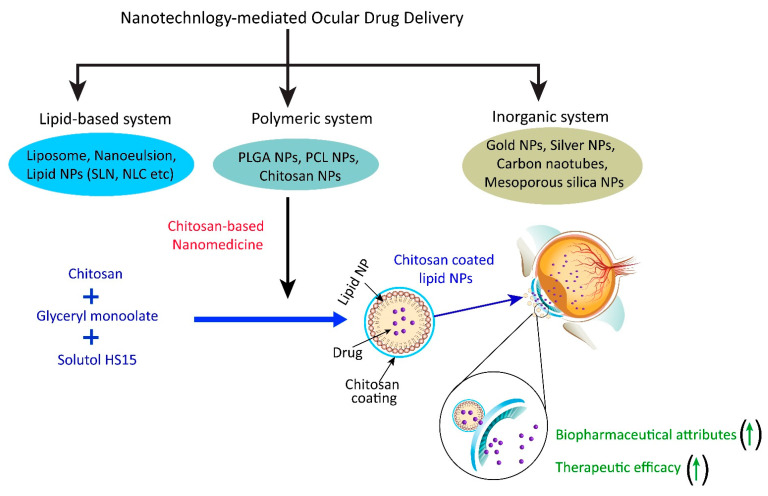
Nanotechnology-mediated drug delivery approach particularly regarding a chitosan-based nanomedicine employed to improve the biopharmaceutical attributes and therapeutic efficacy of a loaded drug in glaucoma. (↑) indicates improvement/increase.

Nanotechnology-mediated drug delivery approaches involved the delivery of loaded therapeutics employing nano drug carriers of polymeric, lipidic, inorganic, and biological origin [11,12]. Nano drug carriers prepared from the polymer entail a nanoparticulate system of natural/synthetic origin and are biodegradable/nonbiodegradable in nature such as chitosan, poly lactic-co-glycolic acid (PLGA), polycaprolactone (PCL), etc. [13,14]. Nano drug carriers prepared from lipids are nanoparticulate/nanovesicular systems of natural/synthetic lipids of a biodegradable/nonbiodegradable nature such as phospholipid (lecithins), stearic acid, glycerol monostearate, compritol^®^, etc. [15,16]. Furthermore, nano drug carriers prepared from inorganic materials are a nanoparticulate system of metallic/nonmetallic origin such as gold, silver, mesoporous silica, carbon, etc. [17,18]. Similarly, erythrocytes (red blood cells) are also utilized as drug carriers for the administration of loaded therapeutics in a biological system to achieve efficacy in different disease conditions [19,20]. Among the various drug carrier systems utilized to improve disease conditions in glaucoma, chitosan is a nanomaterial widely explored for ocular drug administration, particularly in the management of glaucoma [21,22]. The specific characteristics of chitosan (such as mucoadhesion to the cornea, biodegradability in nature, antimicrobial properties, etc.) [23] make it a promising nanomaterial to design a nanomedicine for ocular drug delivery in the management of glaucoma (Schematic illustration in Figure 2). The comparative advantages of nanotechnology-mediated ocular drug delivery with respect to conventional ocular drug delivery are summarized in Table 1.

The present manuscript provides a detailed discussion of the recent advancement of chitosan-based nanomedicine for its utilization to improve the efficacy of loaded therapeutics in better glaucoma management. The manuscript also discusses the significance of chitosan-based nanomedicine for its ocular delivery in glaucoma along with main emphasis on recent research carried out in the last 2 years in this area.

## 2. Significance of Chitosan-Based Nanomedicine to Overcome Drug Delivery Challenges in Glaucoma

Chitosan is a biodegradable natural polymer that has been investigated extensively due to its strong mucoadhesive qualities [24]. The drug’s mucoadhesion and retention time on the ocular surface are enhanced by the ionic interactions enabled by its positively charged nature with the anionic ocular mucosa [25]. As a result, a chitosan-based nanoparticulate system can lessen the number of ocular injections required and boost long-term patient compliance [26]. Chitosan improves permeability by relaxing the tight connections between cells [27]. Furthermore, it is produced from crustacean exoskeletons and fungal cell walls via deacetylation, so its production cost is low and its ecological impact is minimal. Chitosan, in particular, demonstrates remarkable swelling behaviors in a variety of physiological environments, making it a potentially useful platform for research into stimuli-responsive biological delivery systems.

**Figure 3:**
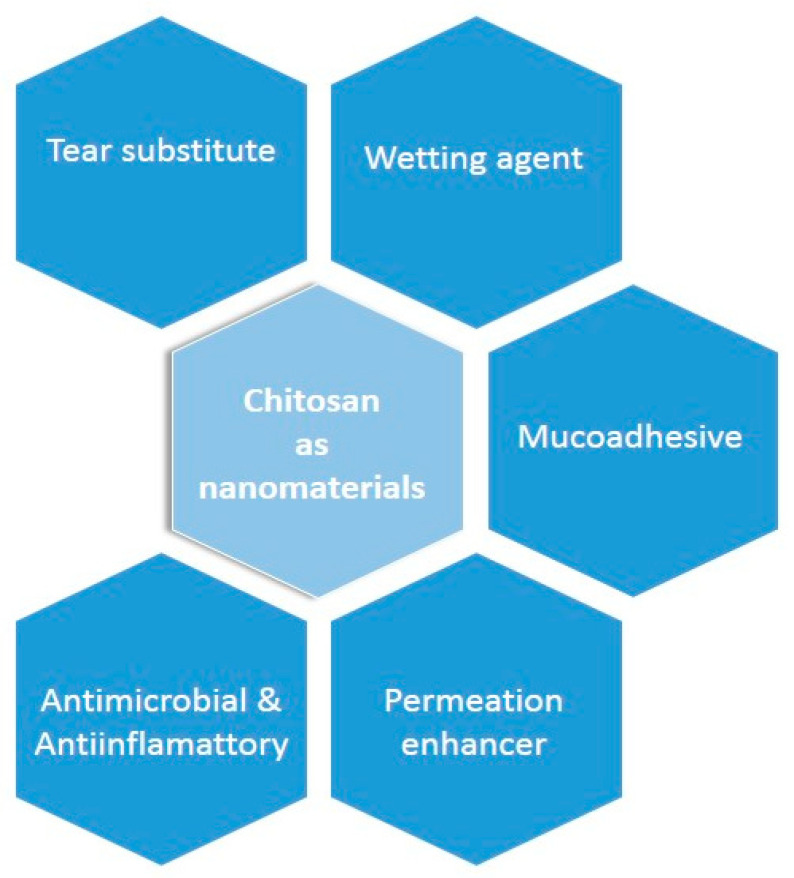
The characteristics that make chitosan a nanomaterial and a promising candidate for nanotechnology-mediated ocular drug delivery in glaucoma.

In recent times, mucoadhesive nanoparticulate systems particularly, chitosan-based nanomedicines, have been widely explored for their specific characteristics (illustrated in Figure 3) in terms of their ocular application to overcome the challenges of ophthalmic drug delivery. It is interesting to note that chitosan immune-modulating capabilities minimize specific inflammatory responses through intracellular signaling pathways (cGAS-STING, and NLRP3) [24]. This signify the possible role of chitosan in the treatment of age-related diseases and its effect on inflammatory cytokines.

**Figure 4:**
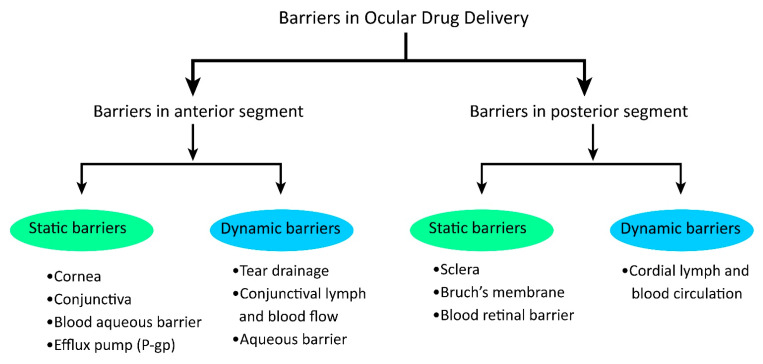
Different barriers in ocular drug delivery for glaucoma pose challenges in ocular bioavailability.

The chitosan-based nanomedicine is helpful in protecting loaded therapeutics from unintended drug release, degradation/instability, and making it easier to cross through different ocular barriers (illustrated in Figure 4) in drug absorption [6].

**Figure 5:**
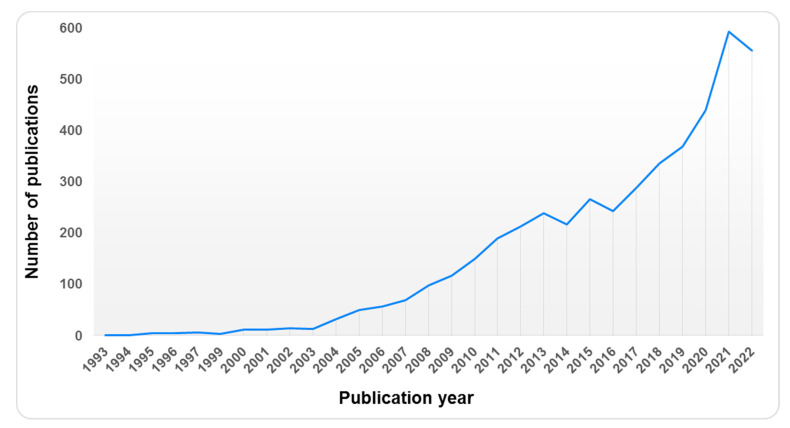
Graph depicts the number of publications in the concerning year published after using keywords “chitosan” and “ocular drug delivery” in the SCOPUS database.

Chitosan-based nanomedicine has a wide utility in biomedical applications including therapeutic, diagnostic, and theranostic purposes in different disease conditions [28]. The literature survey using the keywords “chitosan” and “ocular drug delivery” in the SCOPUS database indicated exponential growth in publications during the last 20 years (1993–2022) as shown in Figure 5.

Furthermore, the analysis of the results showed that out of nearly 4500 publications in the last 20 years, more than 1100 publications had been added to the SCOPUS database in just these two years. Therefore, in the present review, the research papers in these two years that explored chitosan for the ocular delivery of drugs, particularly in the management of glaucoma, were discussed in detail. The different types of chitosan-based nanomedicine employed for ocular drug delivery are discussed in the subsequent section.

## 3. Different Types of Chitosan-Based Nanomedicine for Ocular Application

**Figure 6:**
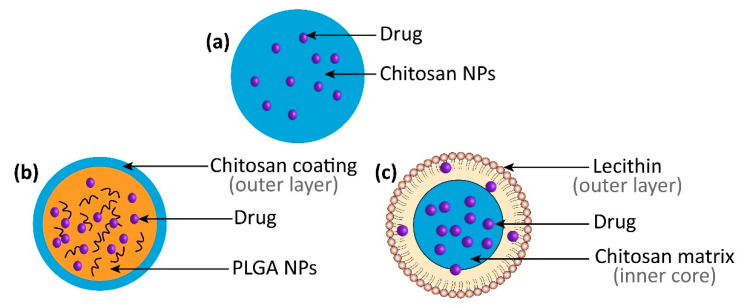
Different types of chitosan-based nanomedicines. (**a**) Chitosan NPs. (**b**) Chitosan-coated NPs. (**c**) Chitosan-based hybrid NPs.

Different types of chitosan-based nanomedicines (such as chitosan NPs, chitosan-coated NPs, and chitosan-based hybrid NPs) have been widely explored in recent years for their ocular applications in glaucoma (Illustrated in Figure 6).

The different characteristics of the chitosan polymer such as molecular weight and deacetylation degree impacted the mucoadhesion to ocular tissues [29]. It is reported in the literature that increasing the chitosan deacetylation degree from 60.7% to 98.5% leads to slower degradation, low drug entrapment, and prolonged drug release profile [30]. Furthermore, chitosan oligosaccharide coated NPs help to delay the clearance of ocular formulation and significantly enhance the AUC of a loaded drug due to improved transcorneal penetration compared to noncoated NPs [31]. The detail related to different types of chitosan-based nanomedicines are discussed in the subsequent section.

### 3.1. Chitosan Nanoparticles

The chitosan nanoparticles are fabricated by ionic or covalent crosslinking, emulsification, precipitation, or combinations thereof [32]. It is a convenient carrier for drugs and is bioactive. Recently, Mohamed et al. fabricated meloxicam-loaded chitosan nanoparticles using the “polyelectrolyte complexation” method [33]. Chitosan (0.25–0.5% *w*/*v*) was first dissolved in an aqueous acetic acid solution (0.5–1% *v*/*v*), and the pH was adjusted to 4.7 using a molar solution of sodium hydroxide. Meloxicam particles were then dissolved in either a tripolyphosphate aqueous solution (0.25% *w*/*v*) or PEG 400 (100% *v*/*v*). Meloxicam-loaded chitosan nanoparticles can be produced spontaneously by adding meloxicam solution drop by drop to a magnetically agitated chitosan solution (10 mL) for 30 min, followed by probe sonication for 10 min.

In another investigation, Ricci et al. prepared chitosan nanoparticles containing indomethacin by the “ionotropic gelation” method [34]. The amine group of chitosan, which has a positive charge, reacts with the sulfonic group of sulfobutyl ether cyclodextrin complexed with indomethacin. The chitosan nanoparticles were stabilized by polysorbate 80 (0.5% *w*/*v*) as a nonionic stabilizer. Furthermore, the prepared nanoparticles were coated with a thiolated derivative of low molecular weight hyaluronic acid. It was also reported in an earlier investigation [35]. The significance of this investigation is that only a small amount of the drug was lost during nano-encapsulation.

### 3.2. Chitosan Coated Nanoformulation System

**Figure 7:**
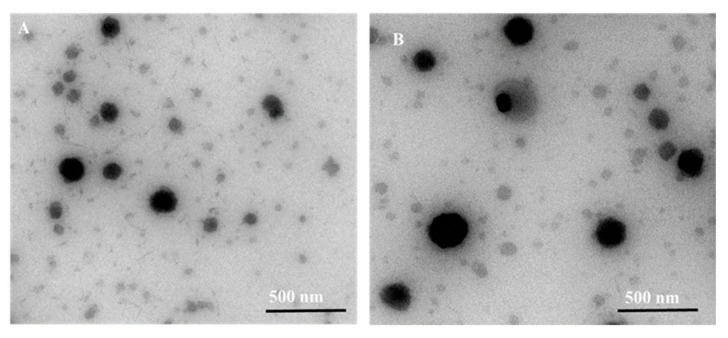
TEM image of (**A**) metoprolol-loaded deformable liposome and (**B**) metoprolol-loaded chitosan coated deformable liposome. Reproduced from Badran et al. [38].

A chitosan coated nanoformulation system was designed by the coating of chitosan over different nanoparticulate-based drug delivery systems such as liposomes, inorganic, polymeric, and lipidic nanoparticles to impart additional physicochemical characteristics (such as improving the residence time, corneal penetration, and ultimately ocular bioavailability of loaded therapeutics) for ocular drug delivery [36,37]. Recently, Badran et al. prepared metoprolol-loaded liposomes coated with chitosan for ocular application [38]. The metoprolol-loaded liposomes were added dropwise to the chitosan solution at different concentrations (0.25–1% *w*/*v*) in an equivalent volume under probe ultrasonication for 3 min, and the resulting suspension was kept on a magnetic stirrer for 2 h at an ambient temperature to achieve successful coating. The study indicated that increasing the amount of chitosan enhanced the vesicle size of liposomes. Moreover, the zeta potential of metoprolol-loaded liposomes was found to change from negative to positive after coating with chitosan. In addition, it was found that the positive charge increased upon increasing the amount of chitosan from 0.25% *w/v* to 1% *w*/*v*. The change in size morphology of metoprolol-loaded deformable liposome and metoprolol-loaded chitosan-coated deformable liposome was examined through transmission electron microscopy (TEM) and shown in Figure 7.

The positive charge could be attributed to the presence of an amine group on chitosan molecules [39]. In another investigation, pilocarpine-loaded ceria nanocapsules were modified with chitosan of different amination levels [40]. Amination levels are critical and may affect the pH-responsive release because free amine groups on the chitosan backbone considerably affect the swelling behavior of chitosan [41].

Ceria nanocapsules werechosen as the carriers for drug delivery because they have a huge cavity inside that can load a significant amount of drugs. It also has strong bioactive properties that help to reduce inflammation, which is a major risk factor for acute glaucoma [42,43]. The rate at which pilocarpine is released from ceria nanocapsules is controlled by the acetylation and deacetylation of the functional chitosan coatings with acetic anhydride and sodium hydroxide, respectively. The surface of the ceria nanocapsule was modified with chitosan using a conjugation method. Briefly, the ends of “phosphonate polyethylene glycol with a carboxylic acid group” can be added to the surface of nanoceria materials so that they can bond with chitosan. Phosphonate groups have a high affinity for cerium surfaces, whereas COOH groups can chemically conjugate with amino groups on the chitosan backbone. The study showed that higher levels of amination can lead to more positive charges on the surface, likely because there are more amino groups [44]. Similarly, chitosan-coated tetrandrine containing bovine serum albumin nanoparticles were formulated and optimized for the concentration of BSA, chitosan, glutaraldehyde, and pH to achieve the desired physiochemical properties for the effective treatment of ocular glaucoma [45]. At pH levels above the isoelectric point (pH > 5) of bovine serum albumin, the net charge of a developed nanoparticle is very negative. This causes molecules and smaller nanoparticles to stick together minimally. The study found that as glutaraldehyde decreased from 8% to 4%, particle size and the polydispersity index decreased significantly, while the zeta potential increased. The coating of chitosan on the nanoparticle is meant to enhance ocular residence and transcorneal penetration of the drug with poor aqueous solubility. The developed system caused the drug to be released over a longer period. Compared to tetrandrine suspension, drug release was much slower in the case of albumin nanoparticles. The drug release was further suppressed by the chitosan coating on albumin nanoparticles. However, the drug release differential between the chitosan-coated albumin nanoparticles and the uncoated albumin nanoparticles disappeared in the later phase, possibly because of water uptake and swelling of the chitosan coat over time [46,47].

### 3.3. Chitosan-Based Hybrid Nanoparticles

Chitosan-based hybrid nanoparticles may be prepared by a single step emulsion-sonication process employing a combination of polymers such as polycaprolactone, hyaluronic acid, polylactic-co-glycolic acid, etc., for ocular drug delivery [48,49]. Recently, Silva et al. prepared epoetin-β loaded chitosan-based hybrid nanoparticles in combination with a hyaluronic acid polymer to improve their mucoadhesion and residence time in the ocular tissues to improve their ocular absorption [50]. Epoetin-β, which is a recombinant form of human epoetin, was chosen as the active ingredient because it might protect and repair nerve cells, which could help to treat glaucoma. Ionotropic gelation was used to make hybrid nanoparticles using different hyaluronic acids. Out of six hyaluronic acids with different molecular weights (50–3000 kDa), one is in crystal form, and another is eye-grade hyaluronic acid. Further research is being conducted on nanoparticles with particle sizes of less than 300 nm, zeta potentials around +30 mV, and a low polydispersity index. It was observed that the high molecular weight hyaluronic acid had the highest entrapment efficiency (39.9 ± 0.6%) and drug loading (18.1 ± 0.3%), respectively. In another investigation, using a quality-by-design (QbD) approach and the ionotropic gelation process, Saha et al. created resveratrol-loaded mucoadhesive lecithin/chitosan hybrid nanoparticles. These nanoparticles were mucoadhesive [51]. Lecithin-chitosan hybrid nanoparticles were made by combining negatively charged lecithin with positively charged chitosan and allowing them to interact with each other to design a hybrid nanoparticulate system. The study utilized poloxamer 407 to dissolve chitosan, while resveratrol was dispersed in an ethanolic solution of lecithin. Subsequently, the alcoholic solution of resveratrol was rapidly injected into the aqueous chitosan-poloxamer 407 solutions under continuous stirring at 1500 rpm to develop chitosan-based hybrid nanoparticles. Chitosan-based hybrid nanoparticles are prepared for various therapeutics including melatonin [52], quercetin [53], insulin [54], diflucortolone valerate [55], and paclitaxel [56].

Different types of chitosan-based nanomedicines (such as chitosan nanoparticles, mucoadhesive chitosan-coated nano drug delivery systems, chitosan-based hybrid nano drug delivery systems, etc.) were explored for ocular applications to improve the biopharmaceutical attributes (such as aqueous solubility, corneal permeability, drug stability, and ocular pharmacokinetic, etc.) and pharmacodynamics performance of loaded therapeutics in glaucoma. The contemporary research carried out in this area in recent times is discussed in a subsequent section.

## 4. Chitosan-Based Nanomedicine for Ocular Application in Glaucoma: Contemporary Research

### 4.1. Improvement in Biopharmaceutical Attributes of Loaded Drugs

**Figure 8:**
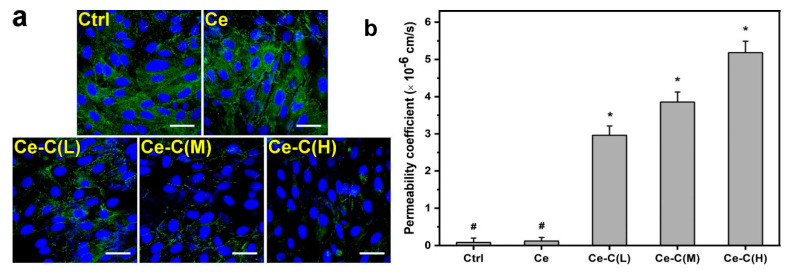
In vitro corneal permeability characteristics. (**a**) CLSM images of SIRC cell layers immunofluorescently stained with DAPI (blue fluorescence) and ZO-1 (green fluorescence) after incubation (for 4 h) with ceria nanocapsule (Ce) and chitosan-coated ceria nanocapsules with varying amination levels [Ce-C(L), Ce-C(M), Ce-C(H)). Ctrl: without test materials. (**b**) The permeability coefficient of ceria nanocapsules and chitosan-coated ceria nanocapsules (Ce) with varying amination levels [Ce-C(L), Ce-C(M), Ce-C(H)). Ctrl: without test materials. Reproduced from Nguyen et al. [40], Elsevier, 2023. * *p* < 0.05 verses all groups; # *p* < 0.05 verses Ce-C(L), Ce-C(M), and Ce-C(H) groups.

Chitosan-based nano formulation systems were investigated for various drugs to improve corneal penetration and residence time on the cornea, thus leading to enhanced ocular bioavailability of antiglaucoma drugs and improving their therapeutic efficacy [57,58,59]. One of the recently published investigations reported that the negatively charged mucus layer on the surface of the eye interacts with cationic chitosan-coated ceria nanocapsules through electrostatic forces [60]. This makes the ceria nanocapsules more resistant to tears and blinks, which means they remain adhered on the cornea surface for a longer period. Chitosan-coated ceria nanocapsules with strong amination diminish negative charges of mucin. The tight connections can be opened with the help of chitosan coatings, as proposed by Nguyen et al. [40], and this ability can be improved by raising the amination level of chitosan. Immunofluorescence labeling of ZO-1 in SIRC cells was used to examine the amination level’s effect on opening the epithelial tight junctions in chitosan-coated ceria nanocapsules. ZO-1 is a crucial cytoplasmic protein involved in membrane activities; it can connect to transmembrane barrier proteins and stabilize tight junctions. At the edges of the SIRC cells, there was a distinct arrangement of ZO-1 in both the control and ceria nanocapsule groups, which showed that Ce-NCs could not open the tight junctions (Figure 8).

As shown in Figure 6, the ZO-1 patterns changed in response to the amination levels of the chitosan coatings for the groups that were treated with low (L), medium (M), and high (H) levels of amination. Chitosan-coated ceria nanocapsules lost pattern integrity and cellular boundaries as amination increased. The result shows that coatings made of chitosan can help ceria nanocapsules to open tight junctions. Furthermore, the ability to open the tight junctions can be increased by adding more amination levels. This study also indicated that the pilocarpine concentrations in the anterior chamber of the eye for the group treated with chitosan-coated ceria nanocapsules with varying amination levels (6.14 ± 2.14 (L), 12.56 ± 1.21 (M), and 25.72 ± 1.68 (H) µg/mL, respectively) were observed to be significantly higher compared to conventional eye drop formulations (0.93 ± 0.64 µg/mL) and the group treated with pilocarpine-loaded ceria nanocapsules (0.58 ± 0.71 µg/mL). The results suggest that the absorption of pilocarpine in the aqueous humor can be enhanced 44-fold by utilizing the chitosan covering with the highest amination level [40].

Tetrandrine shows promise as a prospective glaucoma therapy [61]. However, its restricted ocular bioavailability is a result of its poor aqueous solubility. After 6 h, merely 2.21 ± 0.7% of the tetrandrine suspension had penetrated the cornea. The apparent permeability coefficient for tetrandrine-loaded albumin nanoparticles increased by a factor of 2.3, and the amount of tetrandrine that was able to pass through the membrane increased to 4.72 ± 0.29%. Furthermore, chitosan coating of albumin nanoparticles showed a significant increase (11-fold and 5-fold) in the percentage of tetrandrine permeated compared to tetrandrine suspension and tetrandrine-loaded albumin nanoparticles, respectively. The developed system helps to improve (4-fold and 1.7-fold) the apparent permeability coefficient compared to tetrandrine suspension and tetrandrine-loaded albumin nanoparticles, respectively. The study also indicated a two-times increase in ocular bioavailability of tetrandrine from developed chitosan-coated albumin nanoparticles compared to tetrandrine suspension and tetrandrine-loaded albumin nanoparticles [45]. In another study, trimethyl chitosan-coated lipid nanoparticles significantly prolonged the residence of tetrandrine in tears and enhanced ocular absorption as compared to tetrandrine solution [62]. In addition, when compared to a pure drug solution, the area under the curve (AUC), elimination half-life, and mean residence time (MRT) of the developed system were increased by 2, 3, and 1.67-fold, respectively.

In another investigation, Badran et al. indicated the enhanced penetration of metoprolol from the chitosan-coated flexible liposomes [38]. The enhanced permeability of metoprolol from liposomes could be due to the nanoscale dimension and flexible membrane of liposomes due to the presence of Tween 80 [63]. Furthermore, the cationic nature of chitosan on the liposome surface provided electrostatic interactions and hydrogen bonding with mucin on the ocular surface [64,65]. Hence, it demonstrated better permeation across the cornea compared to uncoated liposomes. In addition, chitosan coating over the surface of various nanodrug carriers was shown to promote corneal permeability by relaxing intracellular or tight connections between corneal epithelial cells [64]. Contemporary research related to chitosan-based nanomedicine utilized to increase the biopharmaceutical qualities of loaded drugs for glaucoma is summarized in Table 2.

### 4.2. Improvement in the Therapeutic Efficacy of Loaded Drugs

Acute glaucoma is often caused by inflammation, and nanoceria is very good at reducing inflammation [70]. Recently, the impact of chitosan coating on the anti-inflammatory characteristics of ceria nanocapsules was investigated [40]. Previous research has shown that this nanoparticulate system helps to remove the free radicals and reduce the generation of inflammatory cytokines such as TNF-α, IL-6, and MCP-1 [71,72,73]. This makes them potent anti-inflammatory agents. LPS control intracellular signaling pathways and were used to cause inflammation [74]. The mitogen-activated protein kinase (MAPK) signaling process can control inflammation by boosting the levels of specific mediators such as Interleukin-6 and Prostaglandin E2, which are common in glaucoma [75,76]. The study showed similar levels of these biomarkers in ceria nanocapsules and chitosan-coated ceria nanocapsules. The investigation also reported that the functionalization of the nanoceria did not affect its ability to fight inflammation. The pharmacological effectiveness of chitosan-coated ceria nanocapsules containing pilocarpine was evaluated in an acute glaucoma model in rabbits. Pilocarpine was used because it makes the ciliary muscles tighten and the pupils constrict, which ultimately lowers the intraocular pressure (IOP) [77]. Notably, the investigation demonstrated that chitosan-coated ceria nanocapsules composed of high amination levels exhibit significant improvements in reducing intraocular pressure in comparison to the marketed pharmaceutical formulation (eye drops of pilocarpine) and uncoated ceria nanocapsules. The drug release profile was maintained for an extended period of time in all chitosan-based formulations. However, only the chitosan-coated ceria nanocapsule with a high amination level was able to reduce and maintain healthy IOP. This is due to the exceptional ability of high-amination chitosan-coated ceria nanocapsules to penetrate the corneal epithelium and lead to improved ocular absorption.

**Figure 9:**
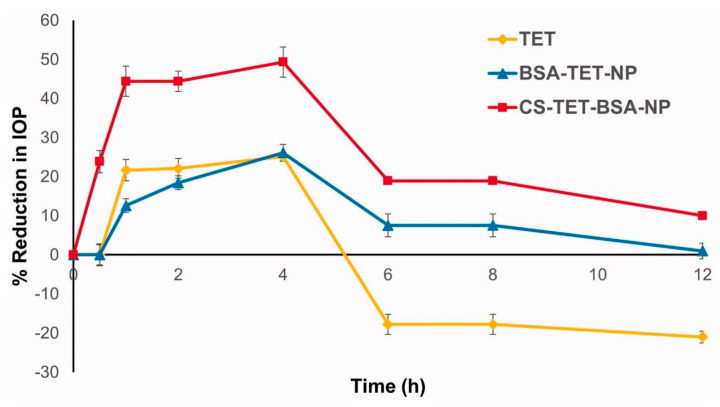
Illustration showed the results of antiglaucoma activity of different treatment investigations (TET: Tetrandrine suspension; TET-BSA-NPs: Tetrandrine loaded bovine serum albumin nanoparticles; CS-TET-BSA-NPs: Chitosan-coated bovine serum albumin nanoparticles containing tetrandrine). It highlights that the coating of chitosan over the bovine serum albumin nanoparticles is further helpful to reduce the IOP in rabbit glaucoma model compared to bovine serum albumin nanoparticles and tetrandrine suspension. Reproduced from Radwan et al. [45], Informa UK Limited, 2022.

Li et al. found in their investigation that tetrandrine can protect ganglionic cells in the retina from the damage caused by ischemia [61]. Research has shown that tetrandrine, at a concentration of 0.3%, reduces intraocular pressure in hypertensive rats. Tetrandrine at a topical dosage of 0.3% was shown to be effective in reducing intraocular pressure [78]. IOP was reduced by tetrandrine suspensions in the period of 0.5–4 h, with a maximum decrease of 25.1 ± 3.8% at 4 h, although this effect was short-lived, perhaps because the drug was rapidly removed from the corneal surface [59]. Tetrandrine-loaded albumin nanoparticles are helpful to reduce the IOP by 26.1 ± 1.08% after 4 h of ocular administration similar to tetrandrine suspension, while chitosan-coated albumin nanoparticles are more helpful to reduce the IOP compared to the tetrandrine suspension and uncoated albumin nanoparticle. Figure 9 presents the reduction in IOP of the eye of a rabbit after a single instillation of tetrandrine suspension, a tetrandrine-loaded uncoated albumin nanoparticle, and/or a tetrandrine-loaded chitosan-coated albumin nanoparticle. It showed a successful reduction in IOP after the instillation of all three types of ocular formulation but the chitosan-coated nano formulation system was more effective in the reduction of IOP compared to other ocular formulations.

As shown in Figure 9, the chitosan-coated nano formulation system is helpful to reduce the IOP by 49.35 ± 2.13% after 4 h of ocular administration. It was observed that the developed delivery system remains effective until 8 h after the ocular administration. It might be because chitosan interactions with mucin facilitate nanoparticle binding to the corneal membrane, extension of the corneal absorption of the drug, and ultimately improvement of the ocular efficacy of loaded therapeutics [79].

In another investigation, Badran et al. evaluated the lowering effect of plain metoprolol, metoprolol encapsulated liposome, and chitosan-coated liposome containing metoprolol on IOP using rabbits as an animal model [38]. The IOP-lowering impact of metoprolol was not fully evident after 1 h of metoprolol-loaded in situ gel instillation, but it was observed after 2, 3, 4, and 5 h following ocular application. In contrast, metoprolol-encapsulated uncoated and coated liposomes incorporated in in situ gels reduced IOP within the first hour after ocular application. Its impact lasted longer than that of a plain metoprolol-loaded in situ gel system. This investigation is in accordance with the previous investigation who reported that metoprolol ophthalmic gels extended the ocular residence time for >5 h [80]. After 6 h of ocular application, chitosan-coated metoprolol containing liposome incorporated in an situ gel system showed a 73.6 ± 4.13% decrease in IOP while metoprolol containing liposome incorporated in an situ gel system showed a 62.3 ± 6.28% decrease in IOP. Moreover, metoprolol-containing in situ gel systems showed only a 54.7 ± 3.15% reduction in IOP after 6 h of ocular application. This sustained effect on lowering IOP is the consequence of the greater corneal permeability of metoprolol upon administration of a chitosan-coated liposome formulation, which may result in increased contact duration and drug retention. Similarly, coating of glyco–chitosan on enalaprilat containing calcium phosphate nanoparticles significantly lowered the IOP for a longer period compared to the pure enalaprilat. The developed enalaprilat nanoparticulate system had a much greater influence on IOP. This effect could be due to the higher zeta potential of glycol–chitosan coated calcium phosphate nanoparticles, which impart a greater affinity towards negatively charged ocular corneal cells and hence provide better penetration [81]. Recently, Rubennicia et al. investigated the IOP lowering effect of latanoprost containing the chitosan–hyaluronic acid hybrid system in albino rats and compared its effect to the latanoprost alone [66]. The study indicated that the developed chitosan-based hybrid system has a significant improvement in the IOP lowering effect compared to that of plain latanoprost.

**Figure 10:**
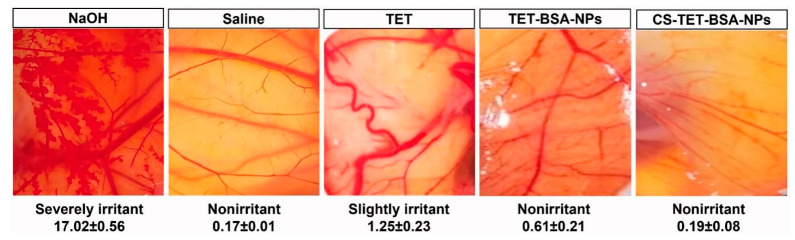
Illustration showed results of the hen’s egg test-chorioallantoic membrane (HET-CAM) investigation after different treatments (TET: Tetrandrine suspension; TET-BSA-NPs: Tetrandrine loaded bovine serum albumin nanoparticles; CS-TET-BSA-NPs: Chitosan-coated bovine serum albumin nanoparticles containing tetrandrine). It highlights that the coating of chitosan over the bovine serum albumin nanoparticles is further helpful to reduce the ocular irritation index value compared to bovine serum albumin nanoparticles and tetrandrine suspension. Reproduced from Radwan et al. [45], Informa UK Limited, 2022.

The neuroprotective effects of epoetin-β in glaucoma are encouraging. Silva et al. prepared a chitosan–hyaluronic acid hybrid system containing epoetin-β to improve their ocular bioavailability through increased mucoadhesion and prolonged residence in the ocular tissues [50]. The study evaluated the possibility of delivering epoetin-β to the ocular tissues through subconjunctival administration. The study found that the designed system could transport epoetin- β to the retina effectively. It was concluded that chitosan-based nanomedicine is thought to be safe for the in vivo system and could be a promising approach to treat retinopathy, such as glaucoma-related optic nerve degeneration. Radwan et al. proved in their investigation that chitosan coating over the nanoformulation system (such as bovine serum albumin nanoparticles) is helpful in further reducing the ocular irritation potential of the nanoformulation system (Illustrated in Figure 10) [45].

The summary of current research on chitosan-based nanomedicines used to augment the therapeutic efficacy of loaded drugs for the management of glaucoma is presented in Table 3.

## 5. Conclusions

The chitosan-based nanoparticulate system indicated promising results in enhancing the biopharmaceutical attributes of various ocular therapeutics through the loosening of tight junctions present on the corneal epithelium. The chitosan nanoparticles or nanoparticles coated with chitosan showed a prolonged release of the drug and also offered mucoadhesion, which helped to augment the residence time of loaded therapeutics in different regions of the ocular tissues. The current review concluded that the chitosan nanoparticles and chitosan coating over different vesicular carrier systems (such as liposomes, micelles, and nanoemulsions) and nanoparticles showing advanced biocompatibility with chitosan, such as mesoporous silica nanoparticles [82], hypercrosslinked polymers [83], and polypeptides [84], have a significant impact to improve the residence time, corneal penetration, and ultimately ocular bioavailability of loaded therapeutics. Moreover, the research showed a significant improvement in the antiglaucoma activity of loaded therapeutics employing chitosan-based nanomedicine in preclinical investigations. However, the literature reveals that the clinical performance of chitosan-based nanomedicines through ocular drug delivery for glaucoma has yet to be addressed in detail. Furthermore, the safety perspectives of the chitosan-based nanomedicine in glaucoma should also be addressed systematically in future studies as it could increase the accumulation of the drug in the ocular tissues for a prolonged period, which could also increase the chances of therapeutic/adverse effects. In-depth molecular mechanisms of chitosan-coated NPs as anti-inflammatories to reduce neuroinflammation should be elucidated in future studies. In addition, ideal physicochemical characteristics (such as the molecular weight, degree of deacetylation, and level of amination) of chitosan being a nanomaterial for drug delivery in glaucoma should also be elucidated in future studies.

## Figures and Tables

**Table 1:** Comparative advantages of nanotechnology-mediated ocular drug delivery in respect to conventional ocular drug delivery.

Conventional Ocular Drug Delivery	Nanotechnology-Mediated Ocular Drug Delivery
Limited aqueous solubility	Improved aqueous solubility
Limited ocular/corneal permeability	Improved ocular/corneal permeability
Immediate effects	Sustained/prolong effects
Nonspecific	Specific
Low bioavailability and intersubject variability	Improved bioavailability and minimized intersubject variability
Limited drug efficacy	Improved drug efficacy
Possibility of untoward effects	Minimized possibility of untoward effects

**Table 2:** Chitosan-based nanomedicine utilized to improve the biopharmaceutical attributes of loaded therapeutics.

Type of Nanomedicine	Therapeutics	Composition	Biopharmaceutical Attributes	Ref.
Chitosan-coated NPs	Metoprolol	Chitosan, phosphatidylcholine, cholesterol	- Developed system exhibited extended drug release and significant mucin mucoadhesion resulting in an increase in residence time after ocular administration. - It has shown a 4.4-fold increase in ocular permeability compared to pure metoprolol.	[38]
Chitosan-coated NPs	Pilocarpine	Chitosan, silica, ethylene glycol, cerium nitrate	- High amination level of chitosan is helpful to enhance the corneal permeability of the developed system by 43-fold compared to medium and low amination levels. - Developed system exhibited a sustained drug release profile.	[40]
Chitosan-coated NPs	Tetrandrine	Chitosan, bovine serum albumin, glutaraldehyde	- Developed system exhibited sustained drug release (19.65% in 2 h) compared to the tetrandrine suspension (35.6% in 2 h). - Corneal permeation profile of the developed system was 23.79% compared to 2.21% for the tetrandrine suspension after 6 h. - Developed system has shown a two-times increase in ocular bioavailability in rabbits compared to the tetrandrine suspension.	[45]
Chitosan-coated NPs	Tetrandrine	Chitosan, glyceryl monooleate, poloxamer 407, kolliphor^®^ HS 15	- Trimethyl chitosan-based hybrid systems exhibited sustained drug release and improvement in pharmacokinetic parameters (AUC_0→∞_, T_1/2_, MRT_0→∞_) compared to the tetrandrine solution.	[62]
Chitosan-based hybrid NPs	Latanoprost	Chitosan, hyaluronic acid, sodium tripolyphosphate	- The developed system may enhance the retention time on the corneal and conjunctiva of loaded therapeutics.	[66]
Chitosan-based hybrid NPs	Epoetin beta (EPOβ)	Chitosan and hyaluronic acid	- Developed system efficiently delivered EPOβ to the retina after administration through the subconjunctival route in immunofluorescence investigation in rats.	[67]
Chitosan-based hybrid NPs	Dorzolamide	Chitosan, polycaprolactone, polyvinyl alcohol	- Developed system exhibited a significant improvement in mucoadhesion to the cornea and an enhancement in permeation across goat cornea compared to dorzolamide solution as a control.	[68]
Chitosan-based hybrid NPs	Brinzolamide	Chitosan, pectin, Tween 80	- Developed system exhibited extended drug release for 8 h and a significant increase in corneal permeability compared to the marketed product.	[69]

**Table 3:** Chitosan-based nanomedicine utilized to improve the pharmacodynamics performance of loaded therapeutics.

Type of Nanomedicine	Therapeutics	In Vivo Model	Pharmacodynamics Performance	Ref.
Chitosan-coated NPs	Metoprolol	Albino rabbits	Developed system exhibited a 73.6% decrease in IOP compared to a 54.7% decrease in IOP by the pure drug in a thermosensitive in situ gel after 6 h of ocular administration.	[38]
Chitosan-coated NPs	Pilocarpine	Acute glaucoma rabbit model	Developed system highly effective in decreasing the extremely high IOP (92 mmHg) to a normal level (20 mmHg) until 4 h of instillation.	[40]
Chitosan-coated NPs	Tetrandrine	Rabbits	Developed system exhibited a 49.35% decrease in IOP compared to a 25.1% decrease in IOP by a pure drug after 4 h of ocular administration.	[45]
Chitosan-based hybrid NPs	Latanoprost	Normotensive albino rabbits	A developed system is more effective in reducing the IOP than by a drug alone.IOP reduction during the treatment period was 27.3% by the developed chitosan-based system compared to 19.3% and 20.3% for the plain latanoprost and marketed product (Xalatan), respectively.	[66]
Chitosan-based hybrid NPs	Brinzolamide	Albino rabbits	Developed system exhibited significant improvement in % decrease in IOP and prolonged IOP lowering effect compared to the marketed product.	[69]
Chitosan-based hybrid NPs	Enalaprilat	Normotensive rabbits	Chitosan-calcium phosphate hybrid system exhibited a significant decrease in IOP after single instillation compared to enalaprilat in solution.	[81]

## Data Availability

Not applicable.
